# A Synthetic Quorum Sensing System Reveals a Potential Private Benefit for Public Good Production in a Biofilm

**DOI:** 10.1371/journal.pone.0132948

**Published:** 2015-07-21

**Authors:** Fang Zhang, Anna Kwan, Amy Xu, Gürol M. Süel

**Affiliations:** 1 Division of Biological Sciences, Section of Molecular Biology, University of California San Diego, San Diego, California, United States of America; 2 University of Texas Southwestern Medical Center, Dallas, Texas, United States of America; Ben-Gurion University of the Negev, ISRAEL

## Abstract

Bacteria predominantly reside in microbial communities known as biofilms, where cells are encapsulated and protected by the extracellular matrix (ECM). While all biofilm cells benefit from the ECM, only a subgroup of cells carries the burden of producing this public good. This dilemma provokes the question of how these cells balance the cost of ECM production. Here we show that ECM producing cells have a higher gene expression response to quorum sensing (QS) signals, which can lead to a private benefit. Specifically, we constructed a synthetic quorum-sensing system with designated “Sender” and “Receiver” cells in *Bacillus subtilis*. This synthetic QS system allowed us to uncouple and independently investigate ECM production and QS in both biofilms and single cells. Results revealed that ECM production directly enhances the response to QS signals, which may offset the cost of ECM production.

## Introduction

**Fig 1:**
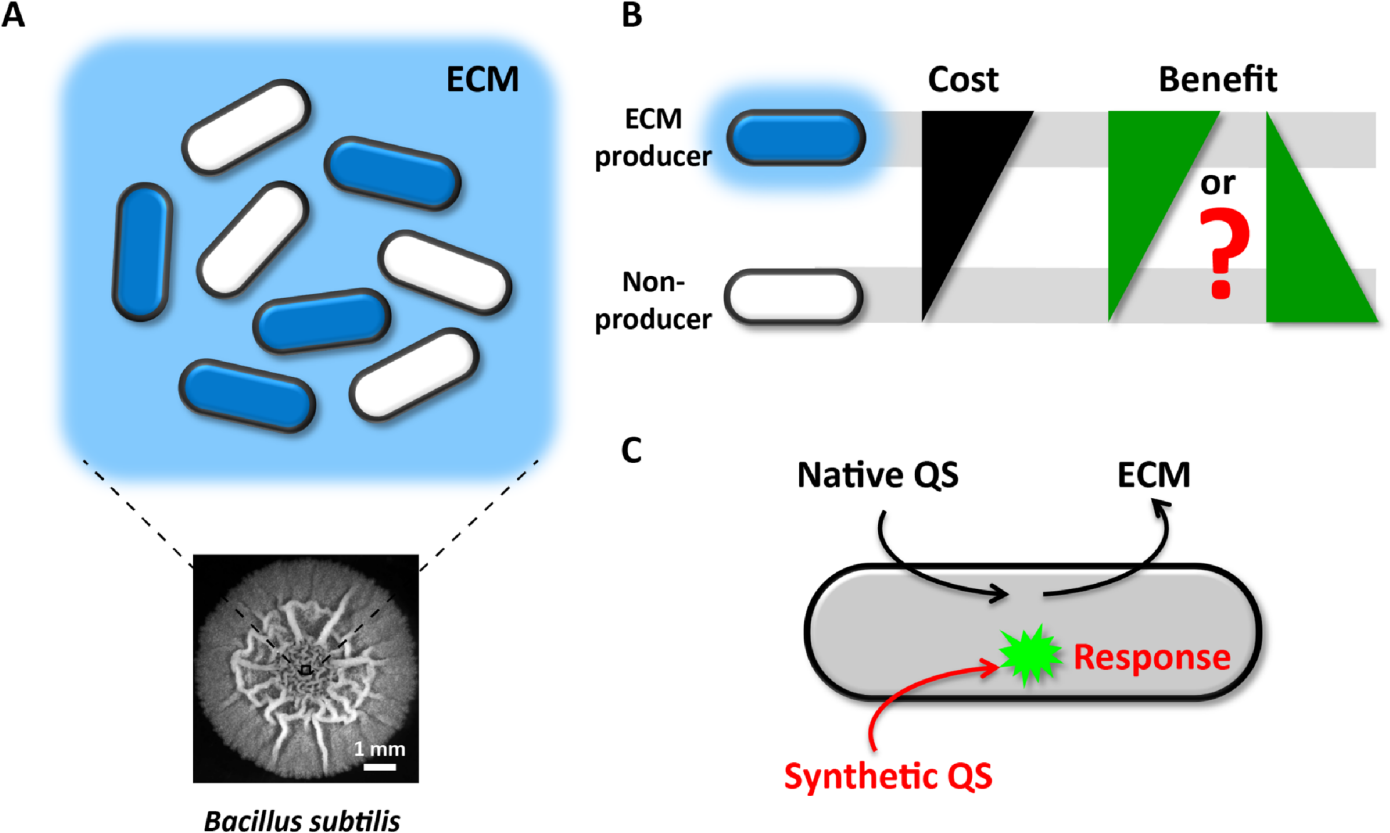
Cost-benefit dilemma for ECM producers in biofilms. (**A**) In biofilms, only a subgroup of cells produces and secretes ECM. However, once secreted, ECM benefits all cells, including non-producers. The bottom picture is the top view of a three-day-old biofilm. (**B**) ECM producers carry a higher cost than non-producers due to production of ECM. Will these producers accordingly get a higher benefit from ECM or not? (**C**) An orthogonal (inert) synthetic QS system was constructed in *B*. *subtilis*. This synthetic QS system does not genetically regulate ECM production, thus enabling us to independently study how the response to QS signals may be affected by ECM production.

Even though bacteria are unicellular organisms, they predominantly reside in structured communities known as biofilms [1,2]. One of the defining characteristics of biofilms is the presence of an extracellular matrix that encapsulates all cells within the community and provides the biofilm with structural integrity [3,4]. The major ECM constituents of most biofilms, for example those formed by *Bacillus subtilis*, are polysaccharides and amyloid-like protein filaments [5–8]. It has been shown that only a subpopulation of cells synthesizes and secretes these ECM components in *B*. *subtilis* biofilms [9,10] (**Fig 1A**). Those ECM producer cells are clearly burdened with a cost at a time of environmental stress and high cell density. This cost is evident by the observation that an ECM-deficient strain can outcompete the ECM producing strain in a mixed culture [11]. However, all cells within the biofilm, even those that do not contribute to ECM production, are believed to benefit from the ECM protection [12–14]. This provokes the question of how the subpopulation of cells that are burdened with ECM production can be sustained within the biofilm community [11,15–19]. It has been argued that expression of ECM by a subpopulation of cells may constitute a primitive form of altruism in bacteria [10] (**Fig 1B**). Here we investigated whether ECM producers could also enjoy a private benefit, countering the cost of this public good production (**Fig 1B**).

How could ECM-producing cells obtain a private benefit? Here we focused on whether ECM expression may affect the response to quorum sensing signals. The response to QS signals can provide a private benefit by inducing intracellular metabolic enzyme expression to better cope with increasing cell density and environmental changes [20–23]. Interestingly, a recent *in vitro* study indicated that quorum sensing molecules could transiently associate with the ECM [24]. Therefore, QS signals may be locally concentrated by the ECM, increasing their availability to cells. However, it is unclear if the transient association of QS signals and the ECM affects the response of ECM-producing cells *in vivo*. Here we investigated whether ECM producing cells may locally concentrate QS signals and thereby obtain a private benefit through an enhanced QS response, by quantitatively measuring a synthetic QS signaling system in biofilm communities and single cells.

ECM expression and QS are tightly coupled and cannot be perturbed independently, since native QS regulates ECM expression [25–31] (**Fig 1C**). Therefore, we constructed an orthogonal (inert) synthetic QS system in *B*. *subtilis* to study how the response to QS signals may be affected by ECM production (**Fig 1C**). This system consists of dedicated “Sender” and “Receiver” cells that can secrete or respond to QS signals, respectively. This synthetic QS system provides the unique opportunity to study QS without interfering with native *B*. *subtilis* QS or ECM production (**Fig 1C**). Investigating synthetic QS in the background of ECM gene deletion strains revealed that ECM expressing cells are more responsive to QS signals. Additionally, we find that the ECM produced by cells retains QS peptides *in vivo*, enhancing the QS response. Finally, we confirm that results from our synthetic QS system also apply to the native QS response of *B*. *subtilis*. Our findings thus support a model where the cellular cost of ECM production can be compensated by an enhanced QS response.

## Materials and Methods

### Strains and growth conditions

All *Bacillus subtilis* strains used in this study were derived from NCIB3610 (a gift from the laboratory of Wade Winkler, University of Maryland, College Park, MD [32]), an undomesticated wild type strain. Strains are listed in S1 Table. Cells were grown in Luria-Bertani (LB) broth (EMD) or on LB agar plates at 37°C. When appropriate, antibiotics were added at the following concentration: 9 μg/mL neomycin (Fisher BioReagents), 5 μg/mL chloramphenicol (Sigma-Aldrich), 300 μg/mL spectinomycin (Sigma-Aldrich), and 100 μg/mL ampicillin (Sigma-Aldrich).

MSgg medium was used when testing cells’ response to AIP. MSgg medium containing 5 mM potassium phosphate (pH 7.0, Fisher BioReagents), 100 mM MOPS (pH 7.0, Sigma-Aldrich), 2mM MgCl_2_ (Fisher BioReagents), 700 μM CaCl_2_ (Fisher BioReagents),50 μM MnCl_2_ (Sigma-Aldrich), 100 μM FeCl_3_ (Sigma-Aldrich), 1 μM ZnCl_2_ (Sigma-Aldrich),2 μM thiamine (Fisher BioReagents), 0.5% glycerol (Sigma-Aldrich) and 0.5% glutamate (Sigma-Aldrich)[8].

### Strain constructions

The *agr* system or fluorescent proteins fused to P*rpsD* were integrated into *B*. *subtilis* using a standard chromosomal integration method. First, target genes were generated through fusion PCR and cloned into chromosomal integration vectors. Then the resulted constructs were confirmed by direct sequencing. The *agr* system was PCR amplified using Phusion high-fidelity DNA polymerase (NEB) and appropriate primers from *Staphylococcus epidermidis* ATTC 14990. Vectors designed for integration into the *gltA*, *amyE* and *sacA* loci were pGlt-Kan (ECE173, constructed by R Middleton and obtained from the Bacillus Genetic Stock Center), pDL30 (a kind gift from Jonathan Dworkin, Columbia University), pSac-Cm [33] (ECE174, constructed by R Middleton and obtained from the Bacillus Genetic Stock Center) respectively. Then, vectors containing target genes were transformed into *B*. *subtilis* using a standard one-step transformation procedure [34]. Strains with targeted integration were confirmed through colony PCR with appropriate primers.

The *ΔepsH* strain is a kind gift from the Roberto Kolter laboratory (Harvard Medical School, Cambridge, MA) [8]. All other strains harboring *epsH* deletion were constructed based on this *ΔepsH* strain. To construct the *ΔtapA operon* strain, upstream and downstream regions of the *tapA* operon were cloned into per449, a generic integration vector constructed for integration into the gene of interest (kind gift from Wade Winkler, University of Maryland, College Park, MD). The resulting vector was then transformed into *B*. *subtilis*.

### Synthesis of *S*. *epidermidis* AIP

*S*. *epidermidis* AIP was synthesized by Protein Chemistry Technology Core in University of Texas Southwestern Medical Center. The synthesis method was previously described by Otto M, *et al*. [35].

### Biofilm formation

The biofilm formation assay is adapted from previous protocols [8]. *B*. *subtilis* strains were grown on LB agar plates with appropriate antibiotics at 37°C for overnight. For each strain, a single colony was picked and grown in 1mL LB liquid culture at 37°C for 6h until saturation. When needed, cultures from different strains were mixed together at a certain ratio and diluted 5000-fold with MSgg medium. Then 1μL culture was spotted on MSgg solid plate (1.5% agar, 3 mm thickness, dried overnight). During the imaging process, cells were incubated at 30°C.

### Microscopy

Movies and snapshots were acquired with an IX71 inverted fluorescence microscope (Olympus), IX83 inverted fluorescence microscope (Olympus) or DeltaVision microscopy imaging system (GE Healthcare). Biofilm movies were taken from the bottom with a 2.5x objective lens (MPLFLN 2.5x/0.08, Olympus) to film the whole biofilm or a 10x objective lens (UPLFLN 10x/0.3, Olympus) to zoom in on certain regions. Images were taken every 40 min. Single cell movies were taken with a 100x objective lens (UPLFLN 100x/1.3, Olympus) while growing 10-fold diluted cell cultures on an MSgg pad at 30°C. Top-view of biofilms was acquired by a Retiga 2000R digital camera (QImaging) via an SZX10 fluorescent stereomicroscope (Olympus).

### Image analysis

ImageJ (National Institutes of Health, http://imagej.nih.gov/ij/) and MATLAB (MathWorks) were used for image processing and analysis. The detailed image analysis methods for biofilms and single cells are described below respectively.

### Biofilm image segmentation and quantification

In a mixed biofilm, regions of each strain were segmented out based on fluorescence images of constitutive markers. P*rpsD* fused with a fluorescent protein (CFP or mCherry) were integrated into each strain except one. For strains containing P*rpsD* driven fluorescent proteins, a fluorescent image was taken accordingly. Then this fluorescent image was processed using ImageJ to determine the region of the strain in two steps. First, the edge was detected using the Otsu method in auto local threshold function. Second, the fluorescence positive region was determined by the analyze particle function. After the region of each fluorescently marked strain was determined, the rest of the area was assigned to the one strain without a fluorescent marker.

After segmentation, the distance of each pixel within Receiver microcolonies to the edge of Sender microcolonies was calculated. In S4 Fig, the average P*3-yfp* fluorescent intensity of all pixels within every 12.9μm (10 pixels) distance was calculated and plotted against its distance from the edge of the Sender microcolony for WT Receiver and *ΔepsH* Receiver, respectively. The average P*3-yfp* fluorescence intensity of WT Receiver and *ΔepsH* Receiver within 12.9μm from the edge of Sender microcolony (grey region in S4 Fig) was calculated and normalized according to the P*3-yfp* fluorescent intensity of WT Receiver. This relative P*3-yfp* intensity of WT Receiver and *ΔepsH* Receiver was measured and the mean was calculated across different movies.

### Single cell segmentation

Single cells were segmented based on phase contrast images using the analyze particle function in ImageJ or image processing and statistics toolboxes in MATLAB, as previously described [36]. When mixing WT Receiver and ECM-deficient Receiver cells together, WT Receiver cells were marked by P*rpsD-cfp*, which enabled us to distinguish between these two cell types.

### Density measurement

Cell density was determined using bright field images as previously described and validated by A. Seminara *et al*. [37]. Relative cell density was defined as–log(I/I_0_), where I is the average image intensity in the region of a certain strain and I_0_ is the average image intensity in regions outside of the biofilm.

## Results

### Construction of a synthetic quorum sensing (QS) system in undomesticated *B*. *subtilis*

**Fig 2:**
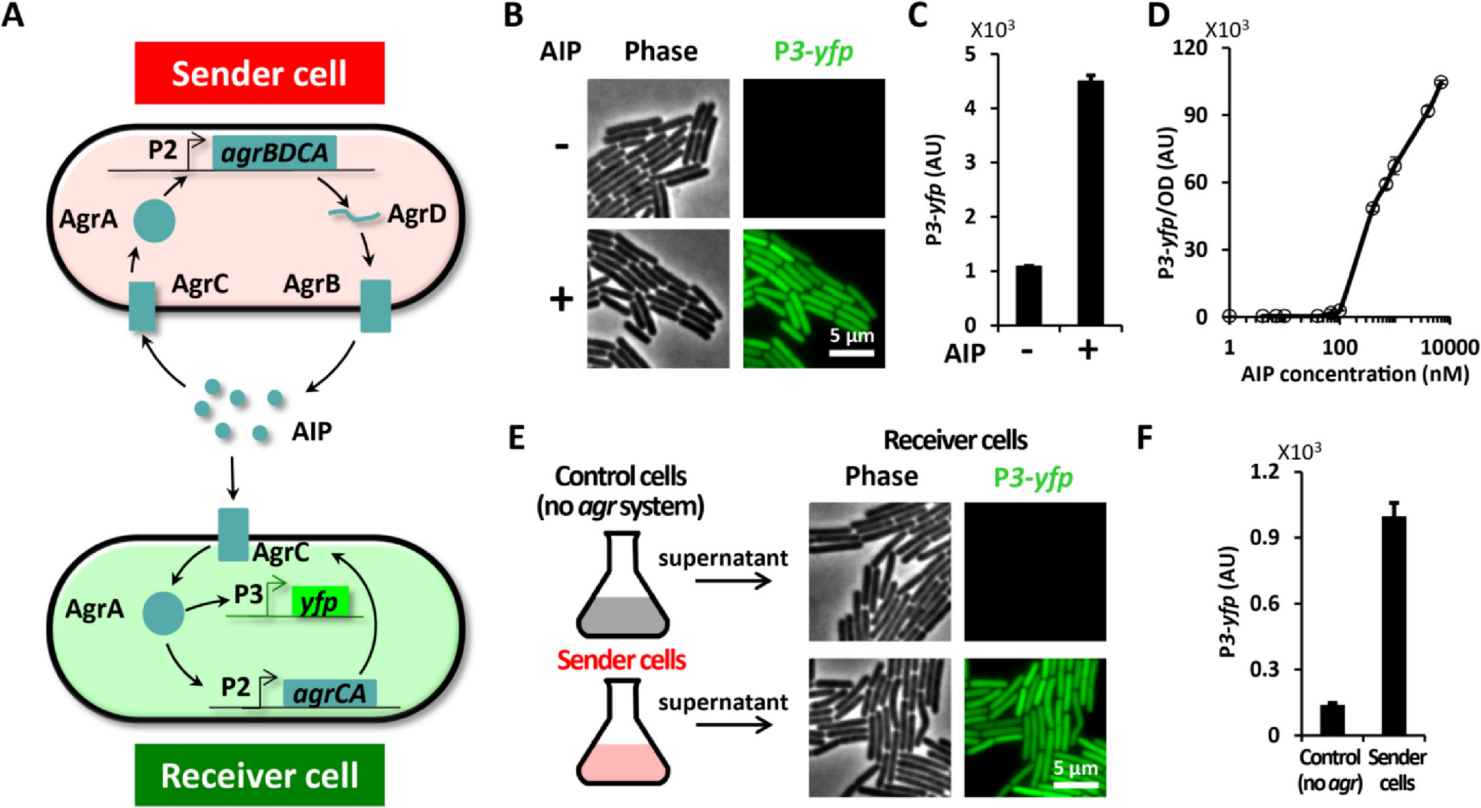
A synthetic quorum sensing (QS) system was built and its functionality and modularity was confirmed in *B*. *subtilis*. (**A**) Diagram of the synthetic *agr* systems in Sender cells (top) and Receiver cells (bottom). The Sender cell contains the entire *agr* operon (P*2-agrBDCA*) and can thus both secrete and sense AIP, the autoinducing peptide. The Receiver cell, on the other hand, carries P*2-agrCA* and P*3-yfp* and thus can respond to AIP by expressing YFP. (**B**) Snapshots of Receiver cells grown on MSgg pads with 0 (-) or 100nM (+) chemically synthesized AIP. *yfp* expression from P*3* is shown in green. (**C**) Mean P*3-yfp* fluorescence intensity from individual Receiver cells with 0 (-) or 100 nM (+) AIP (mean ± SEM, n = 78 cells, *p*<0.0001). (**D**) Dose response curve of Receiver cells to AIP (mean ± SEM, n = 2). (**E**) Images of Receiver cells grown on MSgg pad supplemented with 50% conditioned media from control cells (no *agr* system) or Sender cells. (**F**) Mean P*3-yfp* fluorescence intensity in Receiver cells grown with conditioned media from control cells or Sender (mean ± SEM, n = 97 cells, *p*<0.0001).

To investigate how ECM expression may affect the response to QS signals, we constructed a synthetic QS system. In particular, we transplanted the Auto-Inducing-Peptide (AIP) based QS system (*agr* system) from *Staphylococcus epidermidis* into the biofilm forming *B*. *subtilis* NCIB 3610 strain (**Fig 2A**). We chose the *S*. *epidermidis* QS system because of the following four main reasons: (1) *S*. *epidermidis* is a gram-positive bacteria, which increases the likelihood that this QS system could be functional in gram-positive *B*. *subtilis* cells. (2) The *S*. *epidermidis* QS system is well characterized and simple [38]. It involves only four components (AgrA-D) that are expressed from a single operon. The *agrD* gene encodes the AIP peptide and AgrB is responsible for posttranslational modification and export of AIP [39]. AgrC is the AIP receptor that activates AgrA, the transcription factor that promotes expression of the *agr* operon driven by the P*2* promoter, as well as gene expression of downstream targets driven by P*3* promoters [40,41]. (3) The *S*. *epidermidis* AIP is a short peptide containing 8 amino acids [35] (**S1 Fig**) and is similar in size to the major *phr* encoded QS peptides in *B*. *subtilis* that range in size from 5–6 amino acids [42,43]. (4) There is no known *agr* system in *B*. *subtilis*, suggesting that the transplanted QS system would not interfere with native *B*. *subtilis* QS. Therefore, transplantation of the *S*. *epidermidis* QS system into *B*. *subtilis* provides a unique opportunity to directly investigate how ECM expression may affect the QS response without interfering with native ECM production or QS.

We began by testing the modularity and functionality of the *agr* system after it was transformed into *B*. *subtilis*. Specifically, we constructed two types of synthetic QS systems in *B*. *subtilis*, namely “Receiver” and “Sender” cells (**Fig 2A**). The Sender cells contain the entire *agr* operon (*agrA*,*B*,*C* and *D*) and are capable of sensing and secreting AIP. In contrast, Receiver cells can only respond to AIP, but not synthesize it as they lack the necessary genes. Receiver cells only contain the AgrC receptor and downstream AgrA transcription factor that regulates expression of the P*3* promoter. Therefore, we can use Receiver cells to measure the response to AIP by monitoring expression of a fluorescent protein reporter YFP from the P*3* promoter (P*3*-*yfp*).

### Receiver cells can respond to chemically synthesized AIP

Quantitative fluorescence microscopy measurements confirmed that *B*. *subtilis* Receiver cells containing the synthetic QS system could indeed respond to extracellular AIP (**Fig 2B**). In particular, we find that addition of 100 nM chemically synthesized AIP induced a nearly 5-fold increase in P*3* expression (**Fig 2C**). We also obtained the AIP dose response curve for P*3* expression in Receiver cells (**Fig 2D**). Receiver cells begin to elicit a detectable response to approximately 70 nM extracellular AIP. The response increased with increasing AIP concentrations and surprisingly did not saturate even with addition of 10 μM AIP, a concentration 3 orders higher than the minimal activating concentration. We also performed two control experiments to determine adverse effects of *S*. *epidermidis* gene integrations or AIP peptide addition to *B*. *subtilis* biofilm morphology. Results show that chromosomal integration of *agr* genes into *B*. *subtilis* does not affect biofilm formation (**S2 Fig**). Furthermore, extracellular addition of 10 μM AIP does not alter *B*. *subtilis* biofilm development or morphology, suggesting that native ECM production is not affected by the presence of *S*. *epidermidis* AIP (**S3 Fig**). Together, these data show that the *S*. *epidermidis agr* system in Receiver cells is functional and does not interfere with native *B*. *subtilis* biofilm development.

### Sender cells secrete functional AIP

Next we tested the ability of Sender cells to synthesize and secrete functional AIP. Specifically, Sender cells were grown in LB for 3 hours after which the supernatant was collected and added to Receiver cells (**Fig 2E**). Exposure of Receiver cells to supernatant collected from Sender cells triggered P*3* expression in Receiver cells (**Fig 2E** and **2F**). Therefore, Sender cells secrete sufficient functional AIP to trigger a QS response in Receiver cells. In contrast, supernatant collected from genetically unmodified *B*. *subtilis* cells did not elicit a response in Receiver cells (**Fig 2E** and **2F**). This data also shows that *B*. *subtilis* cells do not express any peptide that can stimulate the *agr* system, further indicating the lack of interference between the synthetic and natural QS systems. Together, these data indicate that the synthetic QS is functional in both *B*. *subtilis* Sender and Receiver cells and performs as intended.

### ECM enhances QS response in biofilms

**Fig 3:**
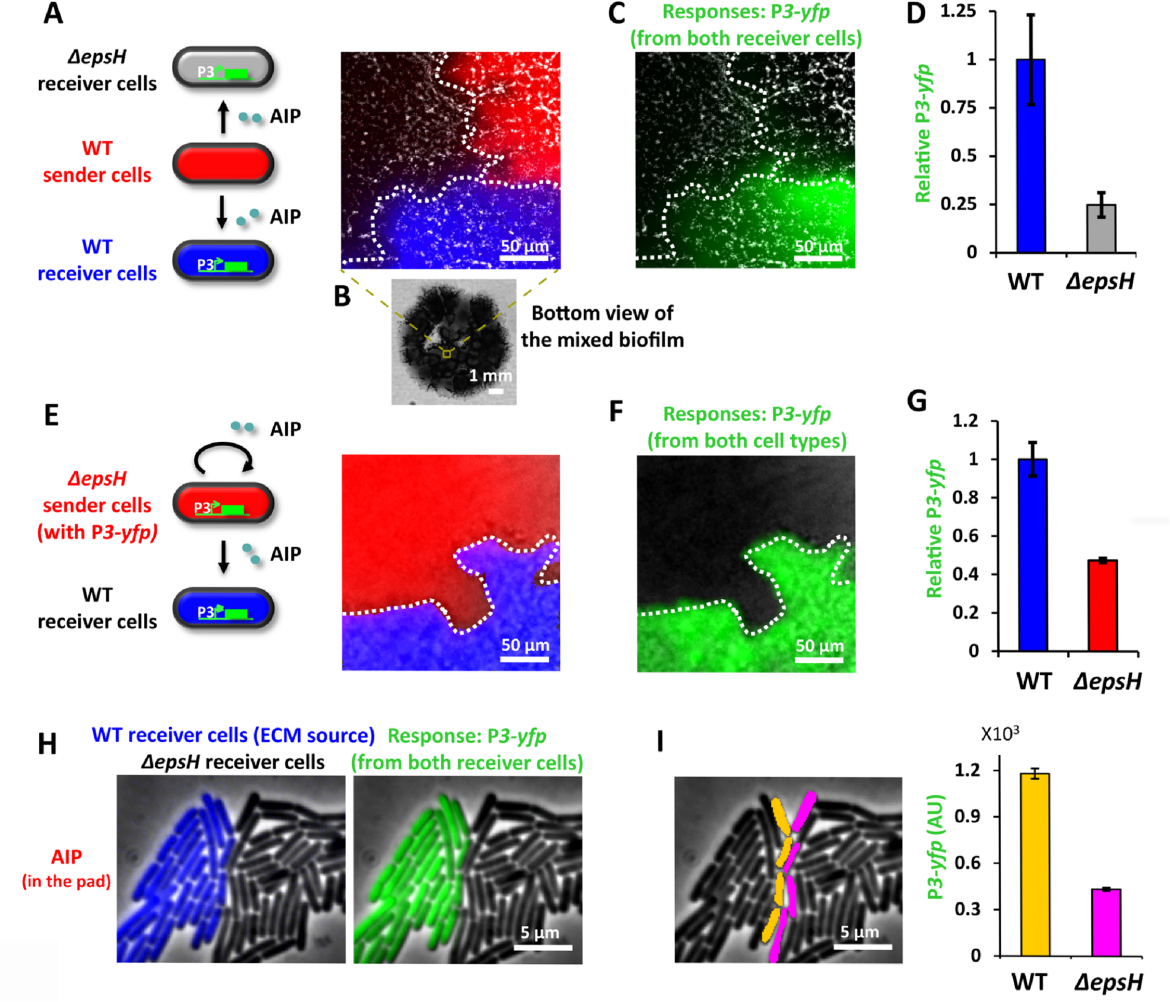
ECM enhances the response of producer cells to QS signal in biofilms. (**A**) A local region in a mixed biofilm where clusters of WT Sender cells (red), WT Receiver cells (blue) and *ΔepsH* Receiver cells (grey) merge. Sender cells are marked by P*rpsD-mCherry* and WT Receiver cells are marked by P*rpsD-cfp*, where P*rpsD* is a constitutive promoter. In this experiment, WT Sender cells (red) secrete AIP that diffuses to nearby WT (blue) and *ΔepsH* (grey) Receiver cells. Both Receiver cells respond to AIP by expressing YFP. (**B**) Bottom view of the mixed biofilm. (**C**) P*3-yfp* overlaid on bright field image showing the same biofilm region as in **A**. (**D**) Relative mean P*3-yfp* intensity of WT Receiver cells and *ΔepsH* Receiver cells (mean ± SEM, n = 3, *p*<0.09). (**E**) A local region in a mixed biofilm where clusters of *ΔepsH* Sender cells (with P*3-yfp*, red) and WT Receiver cells (false colored as blue) merge. *ΔepsH* Sender cells (with P*3-yfp)* are marked by P*rpsD-cfp*. In this experiment, *ΔepsH* Sender cells (red) secrete AIP which induces a response in the *ΔepsH* Sender cells (red) themselves. Meanwhile some AIP will diffuse to nearby WT Receiver cells and trigger a response. (**F**) P*3-yfp* overlaid on bright field image showing the same biofilm region as in **E**. (**G**) Relative mean P*3-yfp* intensity of WT Receiver cells and *ΔepsH* Sender cells (with P*3-yfp)* (mean ± SEM, n = 3, *p*<0.05). (**H**) A snapshot of WT (blue) and *ΔepsH* (grey) Receiver cells growing near each other on an MSgg agar pad containing 100nM AIP. WT Receiver cells are marked by P*rpsD-cfp*. The response, P*3-yfp*, is shown in green on the right panel. (**I**) Response of WT (false colored as orange) and *ΔepsH* Receiver cells (false colored as magenta) on the interface where these two strains merge (mean ± SEM, n = 39 cells, *p*<0.0001).

Next, we investigated directly in biofilm communities whether QS signals would be affected by ECM expression. Specifically, we grew mixed biofilms that contained Sender cells together with two types of Receiver cells, where one Receiver strain was deficient in ECM production (**Fig 3A**). The ECM deficient Receiver strain was obtained by deleting the *B*. *subtilis epsH* gene (**S1 Table**). The *epsH* gene encodes a critical enzyme necessary for extracellular polysaccharide synthesis and it has been shown that *ΔepsH* cells are deficient in ECM production and biofilm development [8]. We grew mixed biofilms that were comprised of adjacent clusters of Sender and Receiver strains (**Fig 3B**). We then measured the QS response of Receiver cells with and without the *epsH* gene to AIP secreted by an adjacent cluster of Sender cells (**Fig 3C**). Results show that the *ΔepsH* strain has approximately four-fold lower QS response (P*3*-*yfp*) compared to the Receiver strain with an intact *epsH* gene (**Fig 3D**). Control experiments show that despite the reduced response to AIP, the *ΔepsH* Receiver strain was still functional and capable of exhibiting an AIP dependent dose response curve (**S5 Fig**). Furthermore, we confirmed that the difference in the response between Receiver strains was not due to differences in cell density (**S6 Fig**). These findings suggest that ECM deficient cells have a reduced response to AIP-mediated QS and are therefore less effective in sensing signals from distant cells.

We asked whether the above-described deficiency in QS response to signals from distant cells would be alleviated if Receiver cells were also directly the source of the AIP signal. Interestingly, we found that even if *ΔepsH* cells are the ones to produce the AIP, the more distant WT Receiver cells exhibited a higher QS response. Specifically, we grew mixed biofilms that contained *ΔepsH* Sender cells and wild type Receiver cells (**Fig 3E**). We find that the *ΔepsH* strain had approximately two-fold lower QS response compared to the wild type Receiver strain (**Fig 3F** and **3G**). Even though *ΔepsH* Sender cells would experience a higher local AIP concentration, distant wild type Receiver cells generated a higher QS response. Therefore, even when the QS signal is not required to travel among cells, ECM deficient cells have a reduced response to locally made QS signals. These data suggest that the higher QS response of ECM producing cells constitutes a private property that is independent of whether cells respond to their own AIP signal or those expressed by distant cells.

### QS related ECM benefit does not extend to directly adjacent cells

To directly test the finding that enhanced QS appears to be limited exclusively to ECM producing cells, we performed experiments at the single cell level. In particular, we wanted to determine if ECM expression could enhance the QS response of directly adjacent cells. We thus turned to experiments in mixed microcolonies that enabled quantitative measurement of the QS response in single cells (**Fig 3H**). Results show that the QS response of WT Receiver cells, capable of ECM production, is clearly higher compared to the response measured in the nearest physically adjacent *ΔepsH* Receiver cells (**Fig 3I**). Furthermore, the low QS response of *ΔepsH* Receiver cells did not decrease further as a function of increasing distance from the WT Receiver cells (**S7 Fig**). Together, these results indicate that enhanced QS response in ECM producing cells is indeed a private property at the single cell level that does not extend to adjacent cells.

### ECM production dependent QS response also affects single cells in liquid cultures

**Fig 4:**
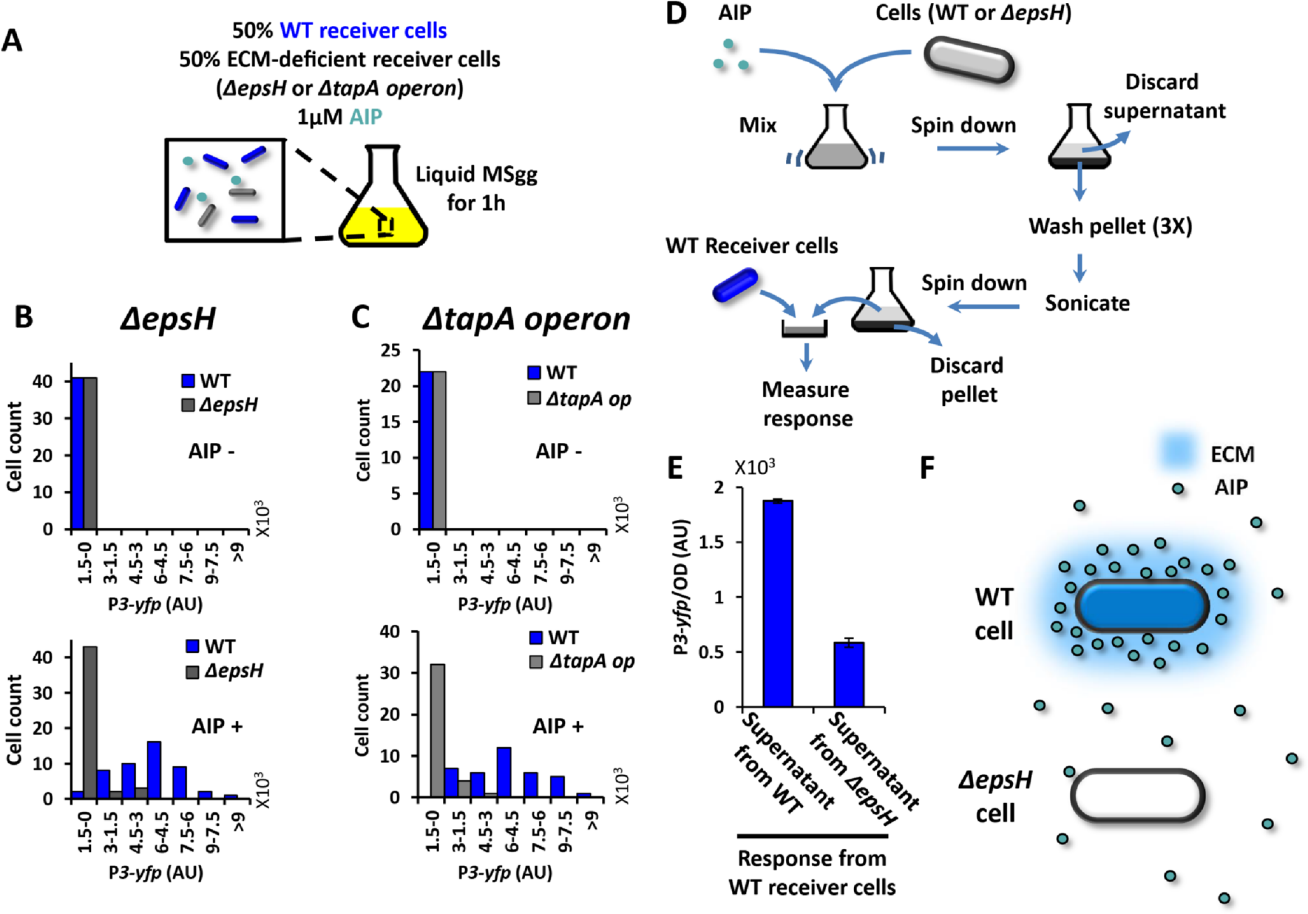
ECM retains AIP around ECM producer cells and enhances their response to AIP even in liquid culture. (**A**) Illustration of experimental setup for B-C. ECM-deficient Receiver cells (*ΔepsH* or *ΔtapA operon*) and WT Receiver cells (P*rpsD-cfp*, blue) are growing together in liquid culture containing 0 or 1 μM AIP for 1h before the response (P*3-yfp*) is quantified. (**B**) Histograms of P*3-yfp* fluorescence intensity from *ΔepsH* Receiver cells and WT Receiver cells (n = 41 cells for the upper panel and n = 48 cells for the bottom panel). The expression of P*3-yfp* was measured for individual cells from microscopic snapshots. (**C**) Histograms of P*3-yfp* fluorescence intensity from *ΔtapA operon* Receiver cells and WT Receiver cells (n = 22 cells for the upper panel and n = 40 cells for the bottom panel). The expression of P*3-yfp* was measured for individual cells from microscopic snapshots. (D) Scheme of the experimental setup in E. (E) Response of WT Receiver cells to AIP extracted from WT cells or *ΔepsH* cells (mean ± SEM, n = 4, *p*<0.0001). (F) A diagram illustrates that WT cells concentrate AIP in their vicinity, while *ΔepsH* cells do not.

Finally we investigated whether the reduced QS response observed in ECM deficient strains was limited to structured communities such as biofilms, or could even be observed in liquid cultures. Accordingly, we grew an equal mixture of wild type and *ΔepsH* Receiver strains for one hour in liquid culture that was supplemented with 1μM of chemically synthesized AIP (**Fig 4A**). We then imaged single cells sampled from this liquid culture. Consistent with our previous results, quantitative fluorescence microscopy showed that ECM deficient *ΔepsH* Receiver cells have a lower response to AIP than wild type Receiver cells, even when grown together in shaking liquid cultures (**Fig 4B**).

Could the reduced QS response be specifically linked to the deletion of the *epsH* gene, or perhaps be a general consequence of ECM deficiency? To address this question, we repeated the same experiment using an equal mixture of wild type and a *tapA* operon deletion strain (**S1 Table**). The *tapA* operon encodes another critical component of the ECM, namely proteins that can form amyloid-like protein filaments [7]. We find that similar to *ΔepsH* Receiver cells, the *tapA* operon deleted Receiver cells also exhibit a reduced QS response compared to wild type Receiver cells (**Fig 4C**). Therefore, regardless of whether the ECM deficiency is caused by lack of exopolysaccharides or amyloid-like protein fibers, the QS response to AIP is reduced in both types of ECM deficient cells. We also ruled out the possibility that the reduced QS response observed in ECM deficient cells was due to a general problem with protein expression by measuring the activity of a ribosomal gene promoter (P*rpsD*) in wild type and ECM deficient cells (**S8 Fig**). Collectively, these results show that the reduced QS response is not tied to the deletion of any particular gene, but appears to be caused by an overall deficiency in ECM production. Furthermore, the reduced QS response in ECM deficient strains not only arises in the context of the biofilm community or microcolonies, but appears to be a single cell level property that even applies in liquid cultures.

### ECM concentrates extracellular AIP in the vicinity of ECM producing cells

Motivated by the recent *in vitro* study that indicated transient physical binding of QS molecules to the ECM [24], we wanted to test whether the elevated QS response we observed was due to local concentration of AIP by the ECM. To determine whether AIP accumulates near ECM producing cells, we added chemically synthesized AIP to wild type cells capable of ECM production. This culture was mixed, spun down and washed three times to eliminate unbound AIP. We then subjected the culture to mild sonication in order to release AIP that may have been retained by the ECM back into the media. In parallel, we subjected *ΔepsH* cells to the same protocol to serve as a control. The supernatant collected after sonication from both WT and *ΔepsH* cells was added to WT Receiver cells and their QS response was measured (**Fig 4D**). We found that WT Receiver cells elicited a higher QS response when exposed to the supernatant from WT cells compared to that obtained from *ΔepsH* cells (**Fig 4E**). This result provides a mechanistic explanation for the enhanced QS response of ECM producing cells, by indicating that the ECM concentrates AIP in the vicinity of cells and therefore increases the likelihood of binding to receptors on the membrane (**Fig 4F**).

### The QS response positively correlates with natural cell-to-cell variation in ECM production

**Fig 5:**
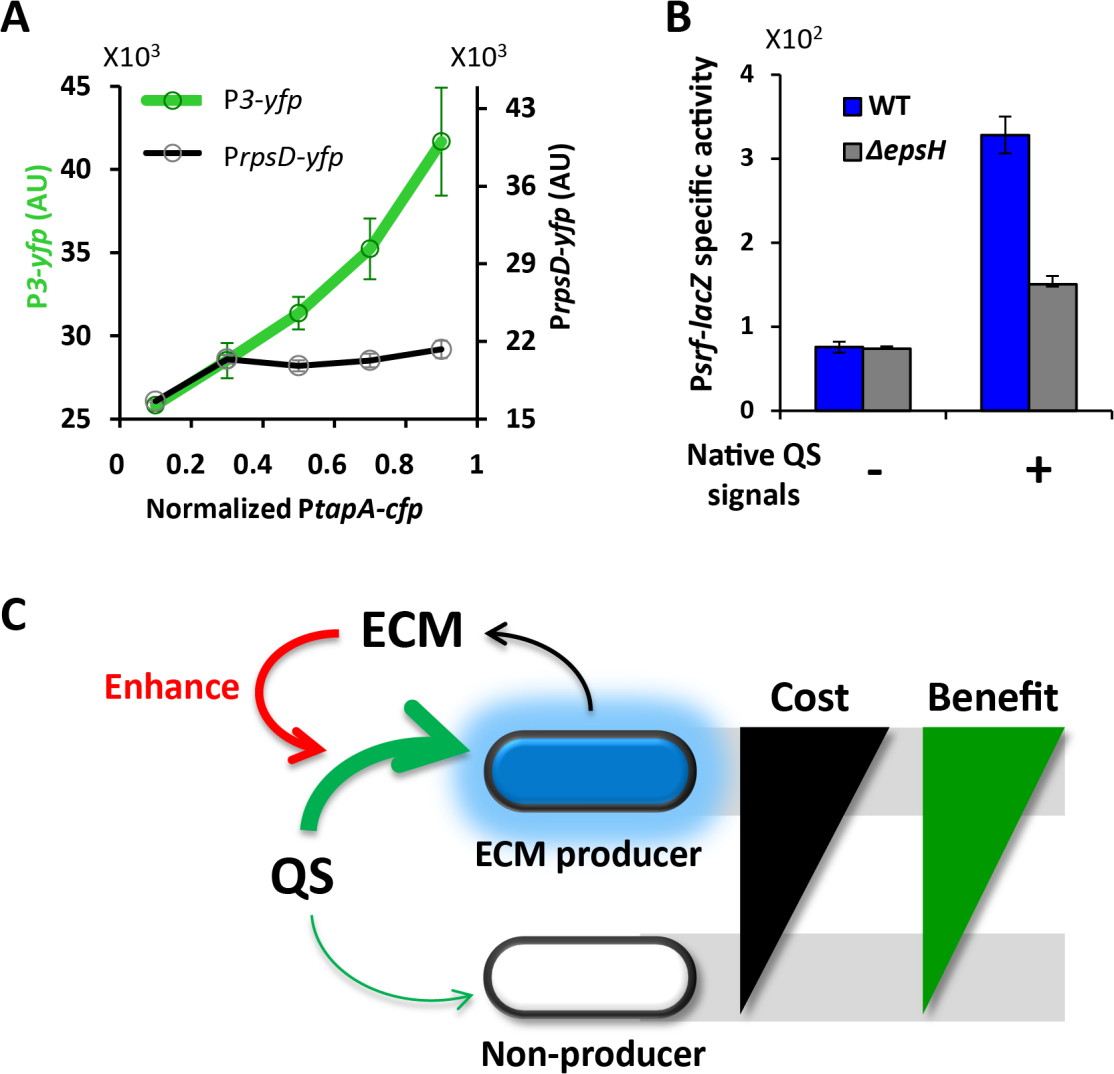
The observation that ECM elevates response applies to native systems. (**A**) Average P*3-yfp* and P*rpsD-yfp* as a function of normalized P*tapA* activity (mean ± SEM, total n = 646 cells). WT Receiver cells containing P*tapA-cfp* were grown in liquid MSgg culture with 1μM AIP. The expression from the reporters P*tapA-cfp* and P*3-yfp* were measured for individual cells from microscopic snapshots. Relationship between P*tapA-cfp* and P*rpsD-yfp* were measured accordingly using the strain containing P*tapA-cfp* and P*rpsD-yfp*. (**B**) Response of WT and *ΔepsH* to conditioned media, which contains native quorum sensing signals that activate P*srf*. Response are measured by P*srf-lacZ* (mean ± SEM, n = 6, *p*<0.01 for native QS signals + group). (**C**) Model: ECM production can exclusively benefit producer cells by enhancing their response to QS, thereby compensating for the cost of ECM production.

The amount of ECM produced by individual cells can vary [9,44]. Therefore, we tested whether the natural cell-to-cell variation that is inherent to ECM production would affect the synthetic QS response. Specifically, we introduced into the Receiver strain a second fluorescent protein reporter to measure the natural cell-to-cell variation in *tapA* expression (P*tapA-cfp*). Single cell analysis of the two-color reporter strain for P*3* and P*tapA* expression shows that the QS response in Receiver cells positively correlates with the natural variation in *B*. *subtilis* ECM expression (**Fig 5A**). In contrast, the control experiment showed that variation in P*tapA*-*cfp* expression did not correlate with expression of the P*rpsD* promoter for a ribosomal gene (**Fig 5A**). Therefore, in addition to the reduced QS response observed in ECM gene deletion strains, response to the QS signal is also affected by the natural variation in ECM expression that is inherent to B. *subtilis*.

#### ECM elevates response to native *B*. *subtilis* quorum sensing signals as well

We asked whether our results obtained using a synthetic QS system would also be relevant to the native QS response of *B*. *subtilis*. Accordingly, we used what is known as conditioned, or spent media that naturally contains QS signals expressed by *B*. *subtilis* cells, such as ComX, and then monitored expression of P*srf*, a *B*. *subtilis* native QS responsive gene promoter [45]. We find that upon addition of conditioned media containing QS signals, WT cells display a higher expression of P*srf-lacZ* compared to *ΔepsH* cells (**Fig 5B**). This result shows that the amplitude of the native QS response also depends on ECM production, indicating that results obtained with our synthetic QS system may be generally applicable to native QS systems in *B*. *subtilis*.

## Discussion

The results presented in this study indicate that the cost of ECM expression may be balanced by an enhanced private response to QS signaling (**Fig 5C**). As the name implies, QS refers to a collective process by which individual cells can sense global population density. While QS contains information about the population, the response to a QS signal is an intracellular process that can be considered “private” to the cell. In fact, recent studies have shown that response to QS can give rise to an advantageous metabolic response in individual cells [20–23]. Therefore, our findings together with results published by other groups suggest a possible private benefit for ECM production. In particular, our data revealed that ECM expressing cells might perceive changes in global cell density earlier and respond accordingly. Earlier detection of an environmental change such as crowding can be beneficial by speeding up stress responses that, for example, induce changes in metabolic activity. Furthermore, ECM deficient cells exhibit a lower response not only to signals produced by distant cells (paracrine), but also self-made signals (autocrine). Therefore, ECM expression appears to promote both paracrine and autocrine mediated QS signaling. Balancing the cost of public good production with a private benefit would allow ECM producing cells to be more easily sustained within the biofilm and also reduce the ability of cheater cells to dominate the community.

The enhanced QS response, however, may not provide a private benefit in all conditions. For example, cells can respond to some QS signals by producing and secreting public goods, such as virulence factors and degradative enzymes [46,47]. If the ECM elevates the response to such QS signals, the response could place an additional burden on ECM producing cells. Furthermore, we note that the relationship between the ECM and QS described here may vary depending on the chemical characteristics and composition of the ECM and QS molecules. As suggested by a previous study [24], hydrophobicity can for example determine the affinity between the ECM and QS signals. If a QS molecule is hydrophilic, rather than attracting it, ECM may even prevent the QS from approaching cells. Therefore, the chemical nature of QS molecules and/or the composition of the ECM may provide bacterial cells with an additional means to tune their QS response.

In addition to serving as a tool to interrogate the relationship between ECM expression and QS, the synthetic QS system we developed here has the advantage of functioning in an undomesticated biofilm community. Therefore, this synthetic QS system can in the future be utilized to investigate various other questions regarding biofilm development. Specifically, biofilm communities are comprised of distinct cell types, and this cellular differentiation appears to be spatially organized. It has been suggested that QS plays a critical role in the spatial organization of cellular differentiation. Therefore, the synthetic QS system presented here may aid in uncovering the principles that govern the spatial organization of cell types within biofilms.

## Supporting Information

S1 Fig Structure of AIP in *Staphylococcus epidermidis* [35]. It has a thiolester linkage between carboxyl-terminus and the middle cysteine.
(TIF)

S2 Fig AIP has no significant effects on B. *subtilis* biofilm formation. WT cells were grown on MSgg plates with 0 or 10 μM AIP for 3 days. Then the bright field image was taken from the top and compared to *ΔepsH* cells grown on MSgg plates for 3 days.
(TIF)

S3 Fig Chromosomal integration of *agr* genes into *B*. *subtilis* does not affect biofilm formation. Four-day-old biofilm structure of control strain (no *agr* system integrated) and Sender strain (with P*3-yfp)*.
(TIF)

S4 Fig Average P*3-yfp* intensity of WT and *ΔepsH* Receiver cells in Fig 3D plotted against the distance from Sender. (TIF)

S5 Fig *ΔepsH* Receiver strain also exhibits an AIP dependent dose response curve. Dose-response curve of *ΔepsH* Receiver cells to AIP (mean ± SEM, n = 2)
(TIF)

S6 Fig Cell densities in regions of the two Receiver strains are comparable. (A) A local region in a mixed biofilm where clusters of Sender cells, *ΔepsH* Receiver cells and WT Receiver cells merge. Sender cells are marked by P*rpsD-mCherry* (red) and WT Receiver cells are marked by P*rpsD-cfp* (blue), where P*rpsD* is a constitutive promoter. This is the same figure as Fig 3B. (B) Bright field image of the same biofilm region as in A. (C) Relative cell density (–log(I/I_0_)) of WT Receiver cell clusters and *ΔepsH* Receiver cell clusters (mean ± SEM, n = 3).
(TIF)

S7 Fig The low QS response of *ΔepsH* Receiver cells did not decrease further as a function of increasing distance from the WT Receiver cells. (**A**) *ΔepsH* Receiver cells grown nearby WT cells (P*rpsD-cfp*, blue) on the MSgg agar pad containing 100nM AIP. *ΔepsH* Receiver cells with different distance from the border of WT cells are false colored with magenta (layer 1), cyan (layer 2) and yellow (layer 3). (**B**) Response (P*3-yfp*) of these three layers of *ΔepsH* Receiver cells (mean ± SD, n = 49 cells).
(TIF)

S8 Fig Fluorescence intensity of P*rpsD-cfp* in *ΔepsH* and WT cells are comparable. (**A**) Snapshot of WT and *ΔepsH* cells grow in MSgg liquid culture. P*rpsD-cfp* is shown in green. (**B**) P*3-yfp* fluorescence intensity in WT and *ΔepsH* cells (mean ± SEM, n = 20 cells).
(TIF)

S1 Table List of strains. (DOCX)

S1 Text Supplementary methods (DOCX)
