# Supplementary figures and images for: A Synthetic Quorum Sensing System Reveals a Potential Private Benefit for Public Good Production in a Biofilm
Source: PLoS One. 2015 Jul 21;10(7):e0132948. doi: 10.1371/journal.pone.0132948 (PMC4510612; doi:10.1371/journal.pone.0132948)

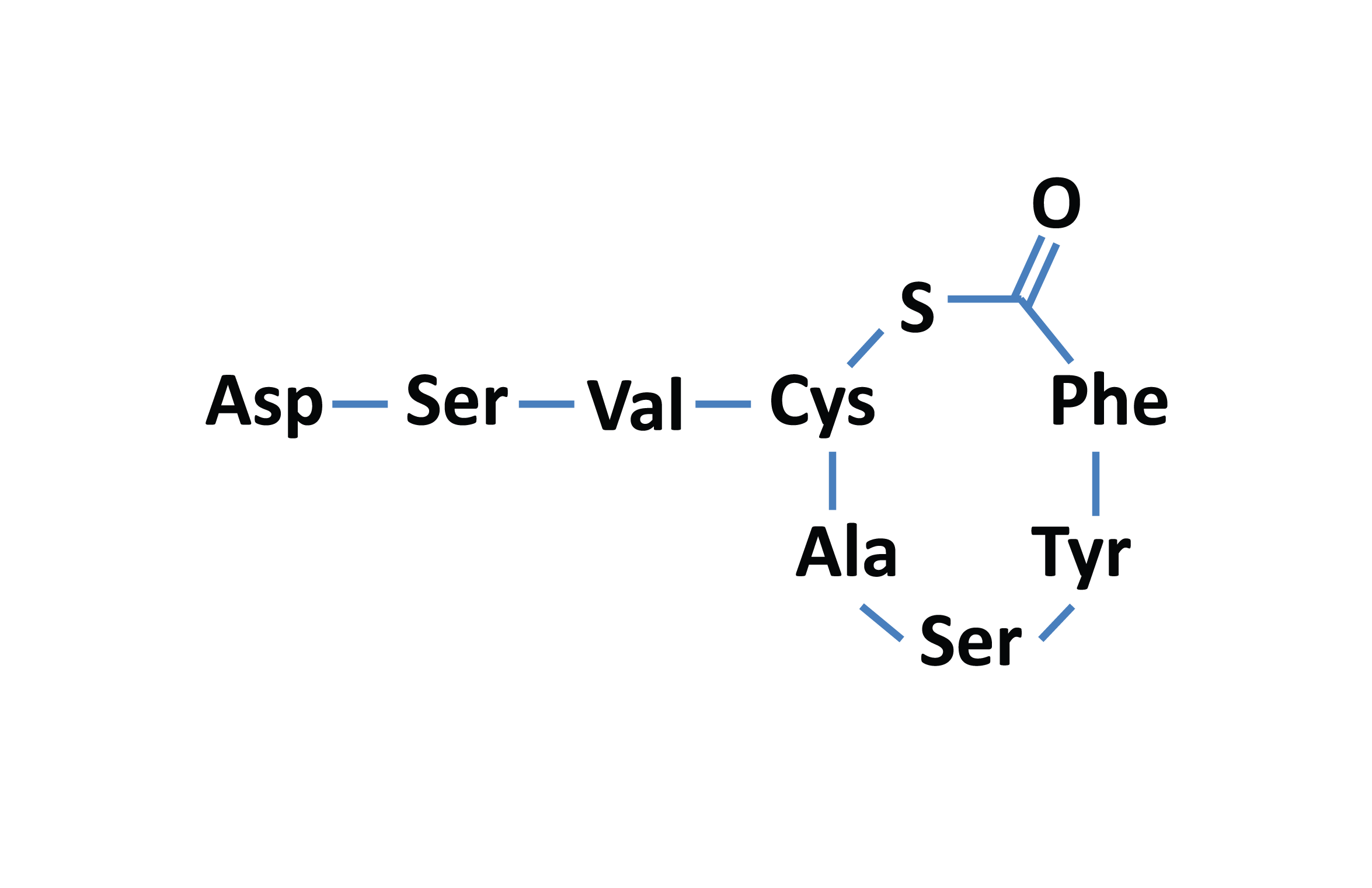

Supplement: S1 Fig — It has a thiolester linkage between carboxyl-terminus and the middle cysteine. (TIF) [file pone.0132948.s001.tif]

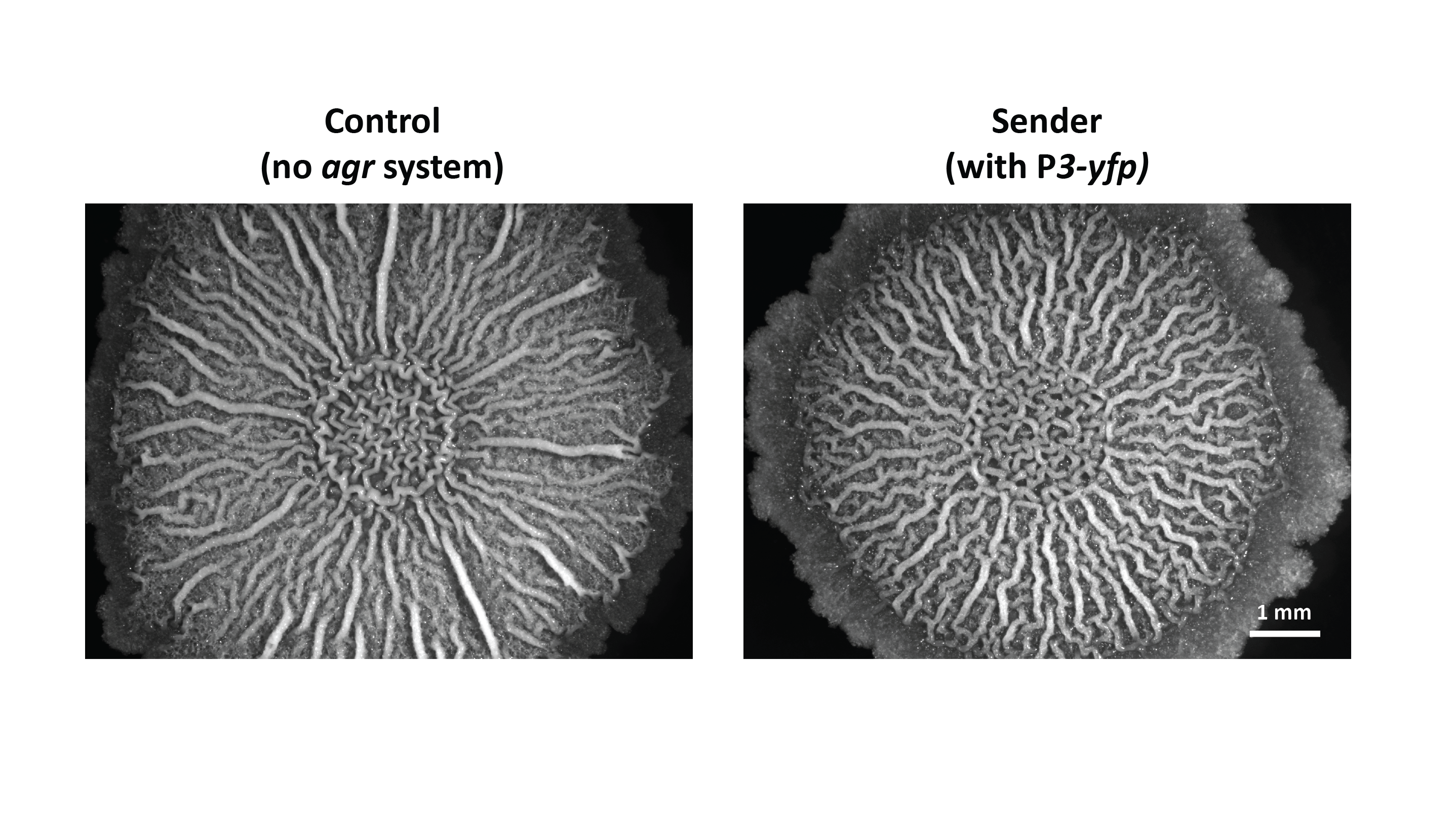

Supplement: S2 Fig — WT cells were grown on MSgg plates with 0 or 10 μM AIP for 3 days. Then the bright field image was taken from the top and compared to ΔepsH cells grown on MSgg plates for 3 days. (TIF) [file pone.0132948.s002.tif]

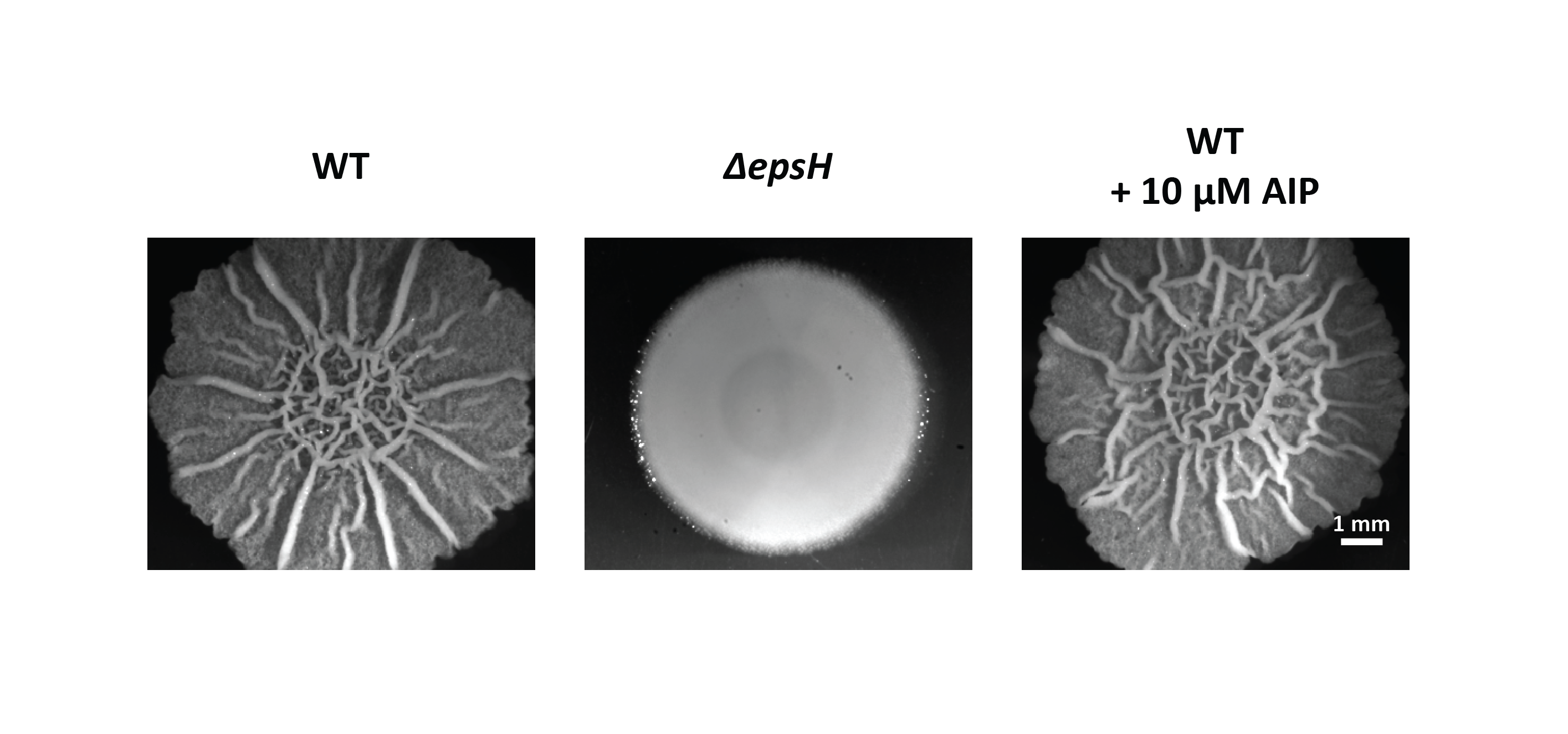

Supplement: S3 Fig — Four-day-old biofilm structure of control strain (no agr system integrated) and Sender strain (with P3-yfp). (TIF) [file pone.0132948.s003.tif]

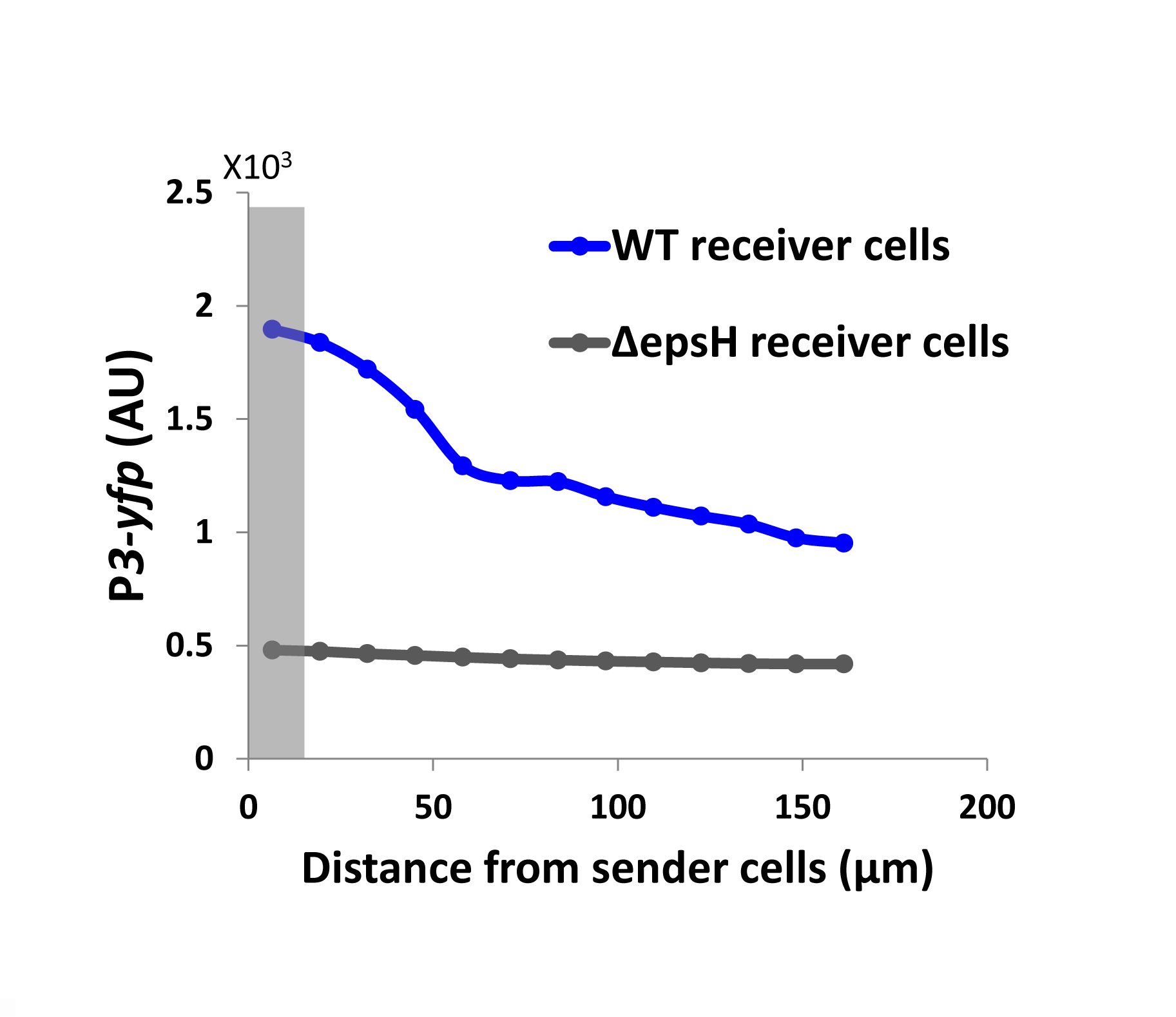

Supplement: S4 Fig — (TIF) [file pone.0132948.s004.tif]

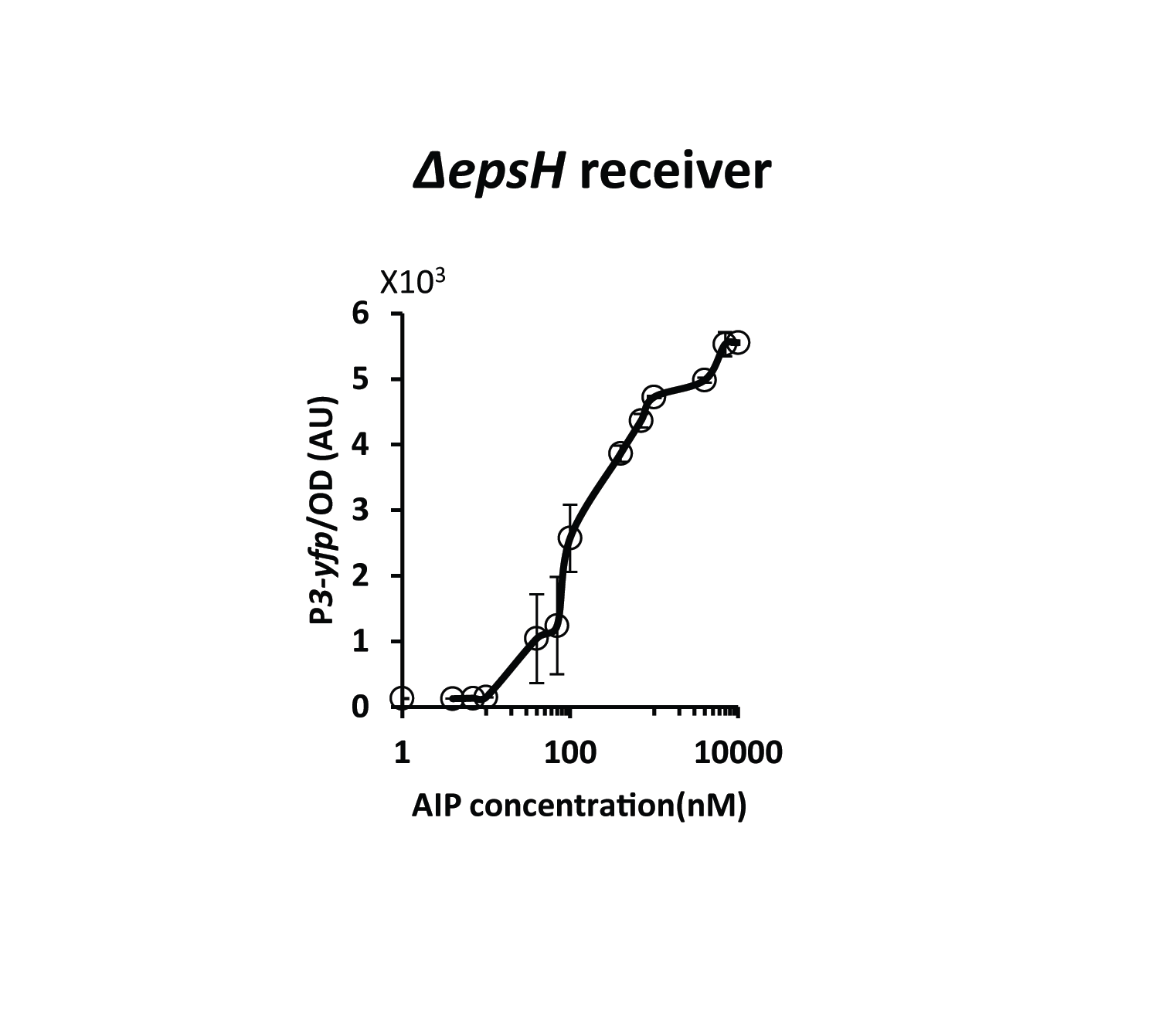

Supplement: S5 Fig — Dose-response curve of ΔepsH Receiver cells to AIP (mean ± SEM, n = 2) (TIF) [file pone.0132948.s005.tif]

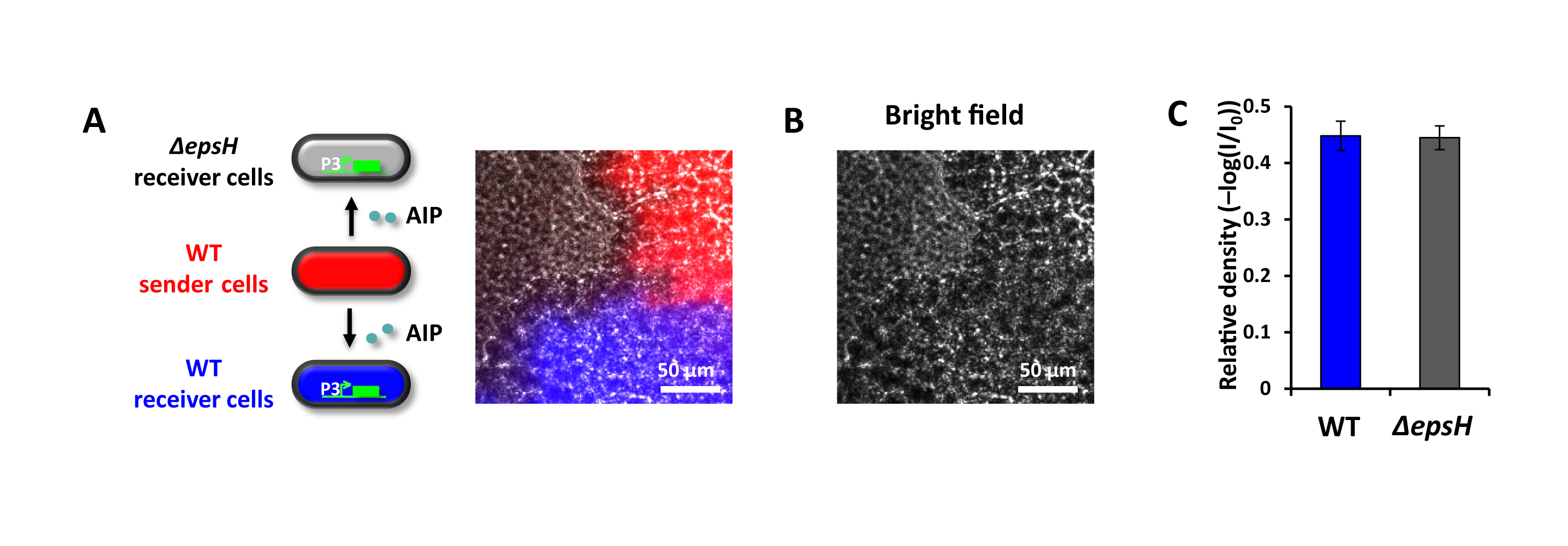

Supplement: S6 Fig — (A) A local region in a mixed biofilm where clusters of Sender cells, ΔepsH Receiver cells and WT Receiver cells merge. Sender cells are marked by PrpsD-mCherry (red) and WT Receiver cells are marked by PrpsD-cfp (blue), where PrpsD is a constitutive promoter. This is the same figure as Fig 3B. (B) Bright field image of the same biofilm region as in A. (C) Relative cell density (–log(I/I0)) of WT Receiver cell clusters and ΔepsH Receiver cell clusters (mean ± SEM, n = 3). (TIF) [file pone.0132948.s006.tif]

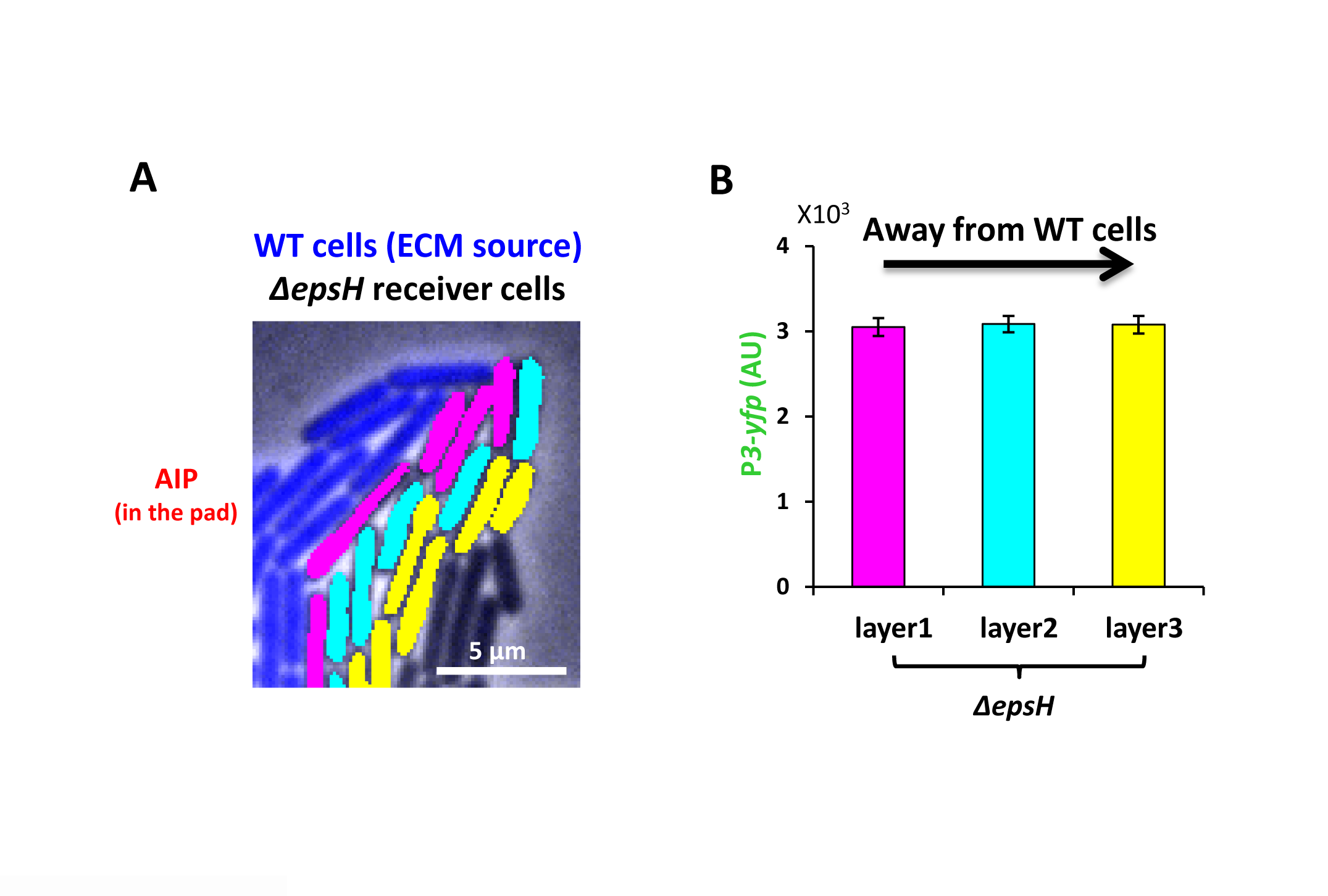

Supplement: S7 Fig — (A) ΔepsH Receiver cells grown nearby WT cells (PrpsD-cfp, blue) on the MSgg agar pad containing 100nM AIP. ΔepsH Receiver cells with different distance from the border of WT cells are false colored with magenta (layer 1), cyan (layer 2) and yellow (layer 3). (B) Response (P3-yfp) of these three layers of ΔepsH Receiver cells (mean ± SD, n = 49 cells). (TIF) [file pone.0132948.s007.tif]

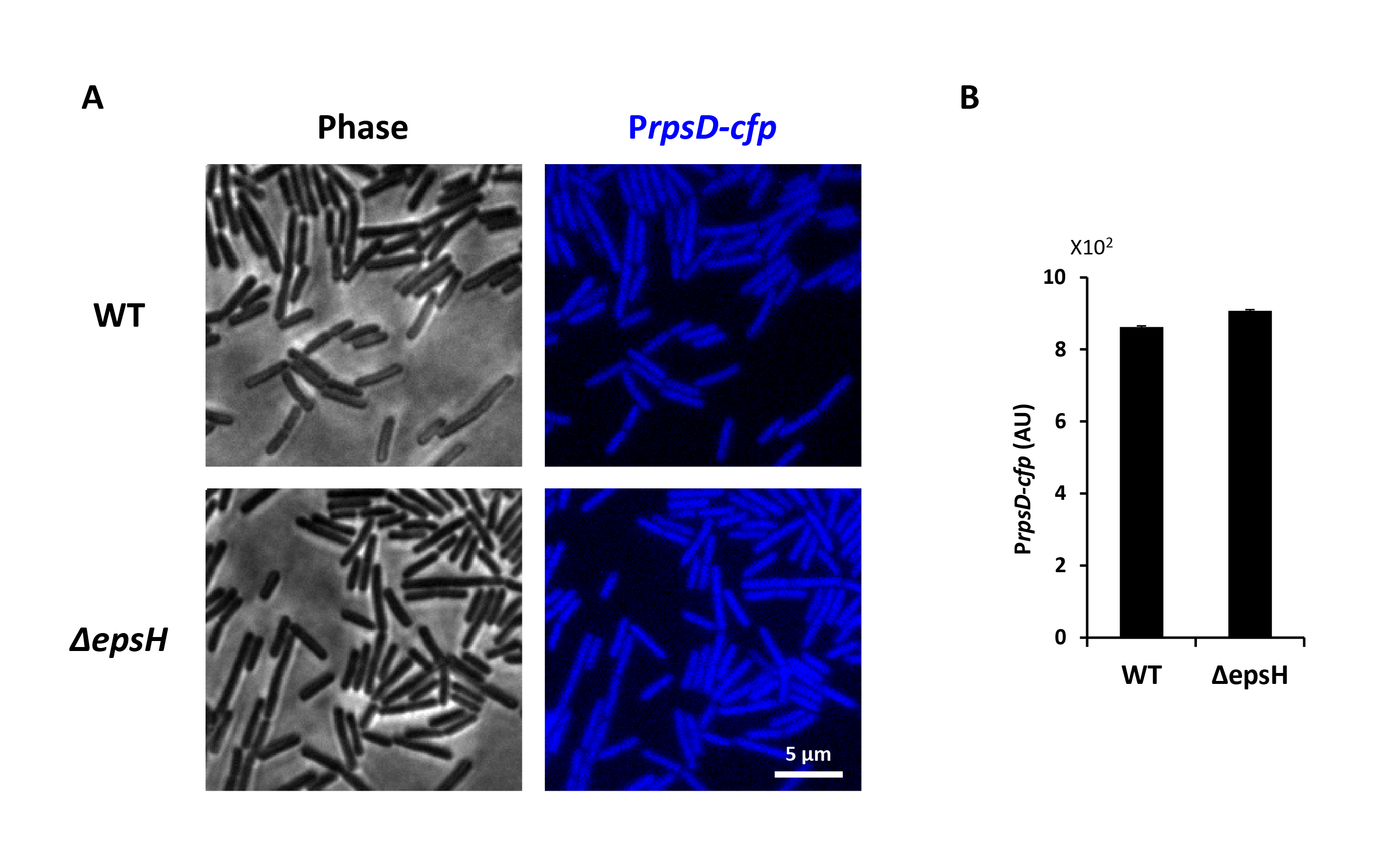

Supplement: S8 Fig — (A) Snapshot of WT and ΔepsH cells grow in MSgg liquid culture. PrpsD-cfp is shown in green. (B) P3-yfp fluorescence intensity in WT and ΔepsH cells (mean ± SEM, n = 20 cells). (TIF) [file pone.0132948.s008.tif]
