# Supplementary material for: A Synthetic Quorum Sensing System Reveals a Potential Private Benefit for Public Good Production in a Biofilm
Source: PLoS One. 2015 Jul 21;10(7):e0132948. doi: 10.1371/journal.pone.0132948 (PMC4510612; doi:10.1371/journal.pone.0132948)
Supplement: S1 Table — (DOCX) [file pone.0132948.s009.docx]

**Supporting information**

**A synthetic quorum sensing system reveals a potential benefit for public good production in a biofilm**

Fang Zhang^1,2^, Anna Kwan^1^, Amy Xu^1^ & Gürol M. Süel^1,*^

**S1 Table. List of strains.**

| Strain | Genotype | Source |
| --- | --- | --- |
| *Bacillus subtilis* | | |
| NCIB3610 |  | a gift from the laboratory of Wade Winkler |
| WT Receiver | *GltA::P3-yfp* (Neo^R^)  *AmyE::P2-agrCA* (Spec^R^)  *SacA::PrpsD-cfp* (CM^R^) | This study |
| *ΔepsH* Receiver | *epsH::tet* (Tet^R^)  *GltA::P3-yfp* (Neo^R^)  *AmyE::P2-agrCA* (Spec^R^) | This study |
| *ΔtapA op* Receiver | *tapA-sipW-tasA::cat* (CM^R^)  *GltA::P3-yfp* (Neo^R^)  *AmyE::P2-agrCA* (Spec^R^) | This study |
| WT Sender | *AmyE:: PrpsD-mCherry* (Spec^R^)  *SacA::P2-agrBDCA*(CM^R^) | This study |
| Sender with optimal AIP yield | PY79  *GltA:: PrpsD-mCherry-PrpsD-spsB* (Neo^R^)  *AmyE:: PrpsD-agrBDCA* (Spec^R^)  *SacA:: P2-agrBDCA* (CM^R^) | This study |
| WT Receiver (no constitutive marker) | *GltA::P3-yfp* (Neo^R^)  *AmyE::P2-agrCA* (Spec^R^) | This study |
| *ΔepsH* Sender (with P*3-yfp)* | *epsH::tet* (Tet^R^)  *GltA::P3-yfp* (Neo^R^)  *SacA::P2-agrBDCA-PrpsD-cfp* (CM^R^) | This study |
| WT Sender (with P*3-yfp)* | *GltA::P3-yfp* (Neo^R^)  *SacA::P2-agrBDCA* (CM^R^)  *AmyE::PrpsD-cfp* (Spec^R^) | This study |
| WT Receiver with P*yqxM-cfp* | *GltA::P3-yfp* (Neo^R^)  *AmyE:: PyqxM-cfp* (CM^R^)  *SacA::P2-agrCA* (Spec^R^) | This study |
| P*rpsD-yfp* P*yqxM-cfp* | *AmyE:: PyqxM-cfp* (CM^R^)  *SacA:: PrpsD-yfp* (Spec^R^) | This study |
| WT with  P*rpsD-cfp* | *AmyE:: PrpsD-cfp* (Spec^R^) | This study |
| WT with  P*srf-lacZ* | *AmyE:: Psrf-lacZ* (CM^R^) | This study |
| *ΔepsH* with P*srf-lacZ* | *epsH::tet* (Tet^R^)  *AmyE:: Psrf-lacZ* (CM^R^) | This study |
| *Staphylococcus epidermidis* | | |
| ATTC 14990 |  | ATCC |
