# Supplementary material for: A Synthetic Quorum Sensing System Reveals a Potential Private Benefit for Public Good Production in a Biofilm
Source: PLoS One. 2015 Jul 21;10(7):e0132948. doi: 10.1371/journal.pone.0132948 (PMC4510612; doi:10.1371/journal.pone.0132948)
Supplement: S1 Text — (DOCX) [file pone.0132948.s010.docx]

**Supporting information**

**A synthetic quorum sensing system reveals a potential benefit for public good production in a biofilm**

Fang Zhang^1,2^, Anna Kwan^1^, Amy Xu^1^ & Gürol M. Süel^1,*^

**Supplementary methods**

**Dose response curve measurement**

Receiver strains were grown on LB agar plates with appropriate antibiotics at 37°C overnight. For each strain, a single colony was inoculated and grew in 1mL plain MSgg liquid medium overnight. The cell cultures were then diluted 100-fold in plain MSgg liquid medium and grew until OD reached 1. 50 μL cultures were added to a clear bottom 96 well plate with pre-added MSgg liquid medium containing increasing concentrations of AIP. The plate was incubated in the shaker for 1h before measuring OD and fluorescence intensity simultaneously using a Infinite 200 microplate reader (TECAN).

**Conditioned media preparation**

The conditioned media preparation method is based on previous work of Magnuson R., *et al.* (1). Wild type *B. subtilis* was grown overnight on plain LB agar plates at 37°C. A single colony was inoculated and grown in 1mL plain MSgg liquid medium for overnight. The cell culture was then diluted 200-fold in plain MSgg liquid medium and grew until OD reached 1.6. After spinning down to get rid of cells, the supernatant was filtered and stored in -80°C.

To optimize AIP yield, the Sender with optimal AIP yield was used in supernatant preparation. As specified in Table S1, the Sender with optimal AIP yield contains two copies of *agr* operon, one is driven by P*2* and the other is driven by P*rpsd*. In addition, this sender contains one copy of *spsB* expressed from P*rpsD*. SpsB is a membrane peptidase that can remove the N-terminal leader of AIP (2), thus facilitating the release of AIP.

**β-galactosidase assay**

Cells were grown on LB agar plates with appropriate antibiotics at 37°C overnight. For each strain, a single colony was inoculated and grown in 1mL plain MSgg liquid medium overnight. The cell cultures were then diluted 200-fold in plain MSgg liquid medium and grown until OD reached 0.1. Mix 1 volume of cell culture with 1 volume of conditioned media and incubate for 1h. Measure and record the OD600 for each samples. Spin down 750 μL cell culture and resuspend the cell pellet in 250 μL Z buffer contains60 mM Na_2_HPO_4_ (Macron Fine Chemicals), 40 mM NaH_2_PO_4_ (Sigma-Aldrich), 10 mM KCl (Sigma-Aldrich), 1 mM MgSO_4_ (BioExpression), 50 mM β-mercaptoethanol (a gift from the laboratory of Kit Pogliano, University of California, San Diego, CA) with pH adjusted to 7.0). Add lysozyme (Sigma-Aldrich) to 0.5 mg/mL and incubate for 10 min at 37°C. Add 0.08% Triton X-100 (AMERESCO) and vortex to lyse the cells. Add 1 another 250 μL of Z buffer and prewarm samples as well as 4 mg/mL ONPG (in Z buffer, a gift from the laboratory of Kit Pogliano, University of California, San Diego, CA) at 30°C for 10min. After prewarm, add 100 μL ONPG into samples and keep them at 30°C until they turn light yellow. Add 250 μL 1M Na2CO3 (Sigma-Aldrich) to stop the reaction and record the reaction time. Measure OD420 of each sample. Specific β-galactosidase acitivity is calculated as OD420 * 1000/ [reaction time (min) * 0.75 (mL) * OD600].

**Single cell imaging**

Response of single cells to AIP was tested in liquid culture containing 1 μM AIP. *B. subtilis* strains were grown overnight on LB agar plates with appropriate antibiotics at 37°C. For each strain, a single colony was inoculated and grew in 1mL plain MSgg liquid medium overnight. The cell cultures were then diluted 100-fold in plain MSgg liquid medium and grew until OD reached 1. Cultures from different strains were mixed together at a certain ratio and incubated with 1 μM AIP. After 1h, 1μL culture was spotted on a solid MSgg pad (1.5% agar, dried overnight) and dried for a while. Pads were then flipped onto glass bottom petri dishes for single cells imaging.

**Promoter definitions**

P*2*: Primers were designed based on the promoter sequence described in a previous study(3) and PCR from *Staphylococcus epidermidis* ATTC 14990. This P*2* promoter is located from 1689507 to 1689972 (direct orientation) on *Staphylococcus epidermidis* ATCC 12228 chromosome.

P*3*: Primers were designed based on the promoter sequence described in a previous study(3) and PCR from *Staphylococcus epidermidis* ATTC 14990. This P*3* promoter is located from 1689610 to 1689810 (complementary orientation) on *Staphylococcus epidermidis* ATCC 12228 chromosome.

P*rpsD*: chromosomal sequence 2853257 to 2853567 (direct orientation) on *B. subtilis* PY79 chromosome. Previous literature has reported this promoter to be constitutively highly expressed(4).

**Supplementary references**

1. Magnuson R, Solomon J, Grossman a D (1994) Biochemical and genetic characterization of a competence pheromone from B. subtilis. *Cell* 77(2):207–16.

2. Kavanaugh JS, Thoendel M, Horswill AR (2007) A role for type I signal peptidase in Staphylococcus aureus quorum sensing. *Mol Microbiol* 65(3):780–98.

3. Otto M, Süßmuth R, Jung G, Götz F, Süssmuth R (1998) Structure of the pheromone peptide of the Staphylococcus epidermidis agr system. *FEBS Lett* 424(1-2):89–94.

4. Jester BC, Levengood JD, Roy H, Ibba M, Devine KM (2003) Nonorthologous replacement of lysyl-tRNA synthetase prevents addition of lysine analogues to the genetic code. *Proc Natl Acad Sci U S A* 100(24):14351–6.
